# Supplementary material for: LncRNA and Protein Expression Profiles Reveal Heart Adaptation to High-Altitude Hypoxia in Tibetan Sheep
Source: Int J Mol Sci. 2023 Dec 27;25(1):385. doi: 10.3390/ijms25010385 (PMC10779337; doi:10.3390/ijms25010385)
Supplement: Supplementary file 1 [file ijms-25-00385-s001.zip › Figure S1.pdf]

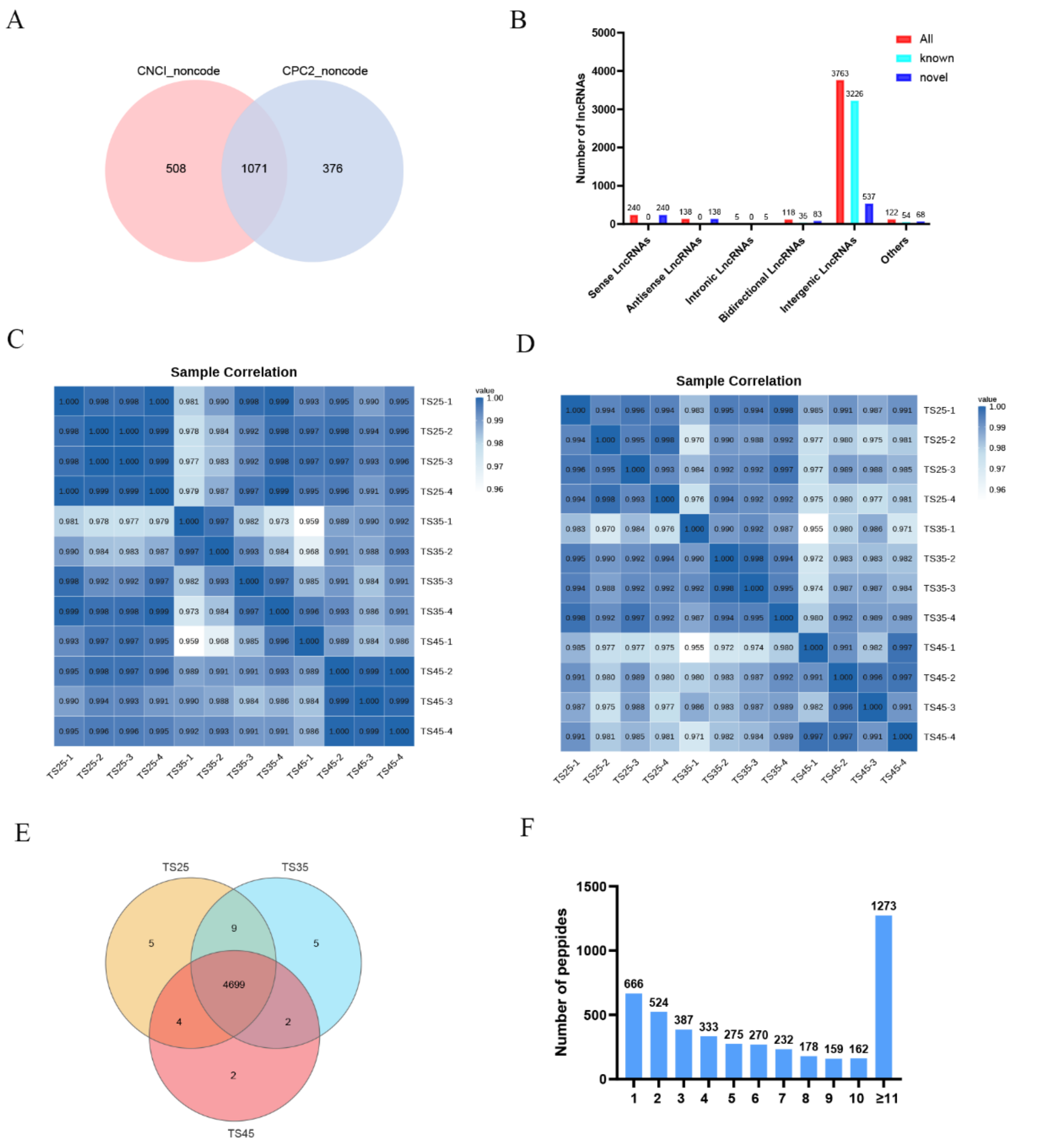

Figure S1. Characteristic analysis of lnc RNAs and proteins in the heart of Tibetan sheep at different altitudes. (A) CNCI_noncode_CPC2_noncode; **(B)** LncRNAs type statistics; **(C)** The correlation of the lncRNA expression profiles; **(D)** The correlation of mRNA expression profiles; **(E)** Venn diagram of proteins in TS25, TS35, and TS45 groups; **(F)** statistical analysis of the number of protein peptide bars.
