# Supplementary material for: LncRNA and Protein Expression Profiles Reveal Heart Adaptation to High-Altitude Hypoxia in Tibetan Sheep
Source: Int J Mol Sci. 2023 Dec 27;25(1):385. doi: 10.3390/ijms25010385 (PMC10779337; doi:10.3390/ijms25010385)
Supplement: Supplementary file 1 [file ijms-25-00385-s001.zip › Table S1.pdf]

Table S1 Differential expression of LncRNA at three different altitudes

Compered group		Lnc RNA symbol			
	Up	ENSOART00020040699	ENSOART00020013993	MSTRG.4748.1	ENSOART00020036454
TS25 vs		MSTRG.3474.4	MSTRG.8419.1	MSTRG.11593.3	MSTRG.7244.2
TS35		MSTRG.5381.3	ENSOART00020007598	ENSOART00020008187	MSTRG.11667.1
		MSTRG.3591.1	MSTRG.14946.1	MSTRG.9775.2	ENSOART00020030306
		MSTRG.14778.4	ENSOART00020008602	MSTRG.1967.2	ENSOART00020023191
		ENSOART00020012333	MSTRG.5118.3	MSTRG.6008.1	
	Down	MSTRG.2891.1	MSTRG.3437.1	MSTRG.6851.1	ENSOART00020005636
		MSTRG.9580.8	MSTRG.16666.3	MSTRG.16669.1	MSTRG.2891.2
		ENSOART00020016244	MSTRG.14712.2	MSTRG.11178.1	ENSOART00020038336
		ENSOART00020019773	ENSOART00020038988	MSTRG.16857.2	MSTRG.11079.1
		ENSOART00020006640	ENSOART00020023776	MSTRG.6851.2	MSTRG.11152.1
		ENSOART00020037755	MSTRG.16115.4	MSTRG.16738.5	
TS35 vs	Up	MSTRG.11461.1	MSTRG.8526.1	MSTRG.12584.1	MSTRG.6375.3
TS45		MSTRG.13743.1	ENSOART00020016099	MSTRG.15337.1	MSTRG.5153.2
		MSTRG.5766.1	MSTRG.4018.1	MSTRG.5765.2	MSTRG.1354.2
		MSTRG.13098.1	ENSOART00020034906	MSTRG.1441.2	MSTRG.7132.1
		MSTRG.1349.1	ENSOART00020005896	MSTRG.10045.1	MSTRG.16409.1
		MSTRG.1.1	MSTRG.13743.2	ENSOART00020030999	MSTRG.362.1
		MSTRG.11178.1	ENSOART00020016687	ENSOART00020030632	ENSOART00020033045
		MSTRG.5938.1	MSTRG.9580.8	MSTRG.6359.8	MSTRG.6654.3
		MSTRG.16092.2	MSTRG.2098.2	MSTRG.5943.1	
	Down	ENSOART00020016122	MSTRG.16222.3	MSTRG.983.1	ENSOART00020024749
		ENSOART00020006729	MSTRG.1193.1	MSTRG.11772.1	MSTRG.15339.1
		MSTRG.1341.1	MSTRG.2891.2	ENSOART00020034936	ENSOART00020007265
		MSTRG.6526.1	MSTRG.4748.1	ENSOART00020036454	ENSOART00020010767
		MSTRG.1967.2	MSTRG.12014.1	MSTRG.15217.3	MSTRG.16732.1
		ENSOART00020013577	ENSOART00020008220	MSTRG.2114.1	ENSOART00020005091
		MSTRG.4037.2	ENSOART00020008223	ENSOART00020036492	MSTRG.14115.2
		MSTRG.5118.3	MSTRG.16647.1	MSTRG.983.2	MSTRG.12489.1
		ENSOART00020040698	ENSOART00020004609	ENSOART00020029824	MSTRG.1877.1
		ENSOART00020002796	ENSOART00020031167	ENSOART00020013655	ENSOART00020012616
		MSTRG.1100.8	ENSOART00020003573	MSTRG.10026.1	MSTRG.14115.1
		ENSOART00020023191	MSTRG.4574.1	MSTRG.11195.1	MSTRG.9775.1
		MSTRG.1100.10	ENSOART00020008273	ENSOART00020000618	ENSOART00020025894
		MSTRG.16347.1	ENSOART00020016070	ENSOART00020036371	MSTRG.5069.1
TS25 vs	Up	ENSOART00020040699	MSTRG.13098.1	MSTRG.7726.1	ENSOART00020006963
TS45		MSTRG.5766.1	ENSOART00020040396	ENSOART00020019309	MSTRG.1259.1
		ENSOART00020013993	MSTRG.11593.2	MSTRG.7916.4	ENSOART00020029432
		MSTRG.13743.1	ENSOART00020006750	ENSOART00020013887	ENSOART00020030306
		MSTRG.9211.2	ENSOART00020024421	MSTRG.13743.2	ENSOART00020001286

	MSTRG.5938.1	MSTRG.5153.2	ENSOART00020039283	MSTRG.1.1
	ENSOART00020023685	ENSOART00020029670	MSTRG.5943.1	MSTRG.10631.1
	MSTRG.3591.1	MSTRG.1349.1	ENSOART00020002028	MSTRG.5381.3
	MSTRG.362.1	MSTRG.10045.1	MSTRG.13236.1	ENSOART00020038924
	ENSOART00020008187	ENSOART00020030440	MSTRG.12760.1	MSTRG.14946.1
	ENSOART00020010355	MSTRG.3416.1	MSTRG.4018.1	
	MSTRG.11593.3	MSTRG.5765.2	MSTRG.4183.2	
Down	MSTRG.6851.2	ENSOART00020023769	ENSOART00020005636	MSTRG.12489.1
	ENSOART00020016122	ENSOART00020040698	MSTRG.6541.2	MSTRG.5820.1
	MSTRG.1341.1	MSTRG.3437.1	ENSOART00020006640	MSTRG.11679.1
	ENSOART00020003962	MSTRG.16666.3	MSTRG.17129.1	MSTRG.11079.1
	MSTRG.1100.8	MSTRG.14963.1	MSTRG.15041.9	MSTRG.15905.1
	ENSOART00020002796	MSTRG.2938.1	ENSOART00020003280	MSTRG.16732.1
	MSTRG.1193.1	MSTRG.16222.3	MSTRG.10057.1	ENSOART00020027412
	MSTRG.2891.2	MSTRG.5672.1	MSTRG.11152.1	MSTRG.1100.10
	MSTRG.2114.1	MSTRG.16669.1	MSTRG.16240.1	MSTRG.10057.2
	ENSOART00020008273	MSTRG.16626.2	MSTRG.12379.1	MSTRG.9242.1
	MSTRG.2891.1	MSTRG.14669.1	MSTRG.9220.2	MSTRG.7424.1
	MSTRG.7055.1	MSTRG.12840.1	ENSOART00020008220	MSTRG.9211.1
	MSTRG.8681.5	MSTRG.983.2	MSTRG.14115.1	MSTRG.13274.2
	MSTRG.1967.2	MSTRG.13274.1	MSTRG.6851.1	MSTRG.16647.1
	MSTRG.10026.1	MSTRG.10112.2	MSTRG.8505.2	ENSOART00020029824
	MSTRG.983.1	ENSOART00020003573	MSTRG.4185.1	MSTRG.11772.1
	MSTRG.16347.1			
