# Supplementary material for: LncRNA and Protein Expression Profiles Reveal Heart Adaptation to High-Altitude Hypoxia in Tibetan Sheep
Source: Int J Mol Sci. 2023 Dec 27;25(1):385. doi: 10.3390/ijms25010385 (PMC10779337; doi:10.3390/ijms25010385)
Supplement: Supplementary file 1 [file ijms-25-00385-s001.zip › Table S2.pdf]

Table S2 Differential expression of proteins at three different altitudes

TS25 vs TS35		DEP symbol			
	Up	MYH11	SERPINA3-8	ACTA1	SHMT1
		MAOB	BGN	MYL9	KRT18
		FLNA	GPX3	CES1	HBB
		SLC4A1	PRELP	SPP2	ALDOB
		SPTA1	Echdc3	RGN	ABCC4
		EPHX2	FABP4	PDLIM7	FAH
		GPX1	ACTN1	ORM1	MUSTN1
		ANK1	NAAA	FKBP5	LTBP2
		CP	COL18A1	INPP1	PSPH
		LAMA5	AKR1C1	CRYM	ITGA3
		ANKRD1	SERPINA3-3	CPS1	Lmod1
		CA4	KRT19	EPB42	ACAT2
		CNN1	LGALS1	CSRP1	Aldh1l1
		PACSIN1	CD109	S100A4	SYPL2
		TXNRD1	THEM4	EHHADH	MGP
		HSPB6	MYOT	GBP3	PCBD1
		Cryzl2	GAA	PGFS	Rdh13
		MFGE8	ACAA1	PLXDC2	ANGPTL2
		HPX	VNN1	CRP	S100B
		HBA1	VNN1	LIPE	AADAT
		HBB	EFEMP1	ATP6V0C	GVINP1
		KRT8	ASS1	OGN	MGRN1
		CA2	MYLK	SELENOM	ACSM5
		CKB	Gstm5	HSD11B1	TEX2
		TAGLN	SMTN	FBP1	PODN
		SOD3	TKFC	CSRP2	APOE
		ADH1C	CLYBL		
	Down	FN1	IGHA1	FETUB	CST3
		APOA4	PNMT	PGLYRP1	NUDT8
		TFRC	TNNI1	APOC3	DTD1
		APOA1	LTF	HMGN2	IGLV2-11
		Acot11	IGLV3-1	CFD	IGLV2-11
		Igkc	C12orf10	C1qc	IGLV2-11
		GCHFR	PRKAG3	SERPINA3-6	IGLV2-11
		PLVAP	C4BPA	CHCHD2	IGLV2-11
		ITIH2	CFP	DPYD	MCRIP1
		A1BG	APOA2	ECM1	MARCHF5
		LDHD	IGLV2-14	CFHR2	IGLV3-19
		CD5L	IGLV2-14	MTMR6	AZU1
		POLR2H	STMN1	STK39	TEX264
		APOH	JCHAIN	MCM7	WDR73
		H1-5	AQP1	MPO	CRYL1

		MYL7	IGLV3-25	ITPA	KCTD10
		GSTT1	MCEE		
TS35 vs TS45	Up	Lamb1	HLA-DRA	H2AC21	CD48
		TFRC	TNNI1	SERPINA6	SYN1
		LMNB1	IDO1	RFTN1	SPARC
		WARS1	SMPDL3B	FRZB	TTL
		ITGA5	REEP5	CELF2	CD74
		ICA	GCHFR	GSTT1	POLR2H
		NIPSNAP3A	Igkc	CFD	MBL
		FSCN1	CDK6	BoLA-DQB	FBN2
		SERPINH1	COX18	TSPO	PRKRA
		STAT1	STMN1	NAF1	IGLV1-40
		FBLN5	GIMAP7	CTSW	IGLV1-40
		LTBP4	CAMK2A	NASP	IGLV1-40
		BPHL	BAK1	NASP	IGLV1-40
		DHTKD1	H1-1	Prkcg	APOM
		H1-5	MTHFR	RBM26	SMCHD1
		SERPINF1	TMEM201	COLGALT1	RAB30
		Coro6	Gimap4	WDR73	RHAG
		IGLV3-25	SCPEP1	CA3	WDR47
		Ahcyl2	CASK	ANP32A	STRIP2
		SAMHD1	CRYL1	PDCD4	MYH1
		H1-0	IFI16	RCC2	MX1
		OLFML2A	Ppp1r1a	LSM5	LIN7B
	Down	MYH11	LGALS3	SELENOF	APOE
		FLNA	TPP1	SAA4	KRT18
		MAOB	VNN1	COL12A1	ALDOB
		EPHX2	VNN1	SELENOM	PSPH
		GPX1	MSRA	A1m	PGPEP1
		TXNRD1	CKB	THBS1	ITGA3
		ADH1C	CLU	S100A1	ANXA8
		SDR39U1	NAAA	INPP1	CES1
		ASPN	SOD3	F9	ADAMTSL5
		CAT	ACTN1	DPY19L1	CRYM
		MYL7	ACTA1	SELENOO	CTSF
		TAGLN	AOC1	LIPE	ELMOD1
		KRT8	AK4	ATP6V0C	PTGIS
		CTSD	KLHL31	PECR	Ntn4
		GPX4	PTGR1	S100A4	PODN
		HPX	PRELP	MAP1LC3A	TNC
		AOX1	ORM1	Aldh1l1	PRXL2B
		MFGE8	OGN	CPS1	HBB
		CNN1	Srm	EHHADH	RBM3
		BLVRA	PDLIM7	CSRP2	ACTG2

		ITIH4	FAH	PDLIM3	MVK
		FABP4	ASS1	ACAT2	SMG1
		GPX3	HEBP1	THA2	FAM53C
		LGALS1	MYLK	TNXB	ACOX3
		BGN	PPT1	Patr-A	RECK
		SERPINB2	Rbbp9	Lmod1	PTP4A1
		SERPINA3-7	MYL9	PAPLN	TMEM109
		PACSIN1	CRP	PTER	LECT2
		ECI1	TACO1	NPC1	MYOT
		NDUFAF7	LUM	PGFS	FUOM
		SERPINA3-3	THEM4	SURF1	DPT
		SERPINA3-8	RGN	EBP	CSRP1
		TNXB	CA4	FBLIM1	KRT19
		OAT	MUSTN1	PSAT1	
TS25 vs TS45	Up	SLC4A1	EFEMP1	SMPDL3B	VAT1L
		SPTA1	HLA-DRA	BAK1	HLA-A
		LMNB1	ITIH5	SCAMP2	Patr-A
		WARS1	H1-5	MTHFR	CASK
		ICA	SERPINH1	SMTNL2	BoLA-DQB
		ADSS1	RIDA	Gimap4	PYCARD
		LTBP4	RIDA	COX18	PRKRA
		NIPSNAP3A	RIDA	ABCC4	Rdh13
		ITGA5	SPP2	H1-1	SPARC
		DARS2	TMEM65	NME4	GVINP1
		SAMHD1	PDXK	TSPO	IGLV1-40
		CA4	OLFML2A	HLA-DRB1	SPARCL1
		FSCN1	Rrbp1	SCPEP1	FRZB
		BPHL	REEP5	MYOT	SYPL2
		ADK	GIMAP7	CD74	EEF1AKMT1
		ANKRD1	Cryzl2	RHAG	FABP7
		FBLN5	LTBP2	TMEM201	CTSW
		GAA	IDO1	CA3	MYL1
		PGM2	GBP3	IGFBP5	SAT2
		CA2	S100B	ABCC4	MGP
		HSPB6	Abcb6	ANP32A	CPT1A
	Down	GPX1	AOC1	TPP1	RBM3
		MAOB	ASAH1	DPY19L1	ELOVL1
		ALDH1A1	AK4	SAA4	TFF2
		SDR39U1	COL6A6	MAP1LC3A	SELENOO
		AOX1	KRT8	ORM1	APOA2
		MYL7	AQP1	CD5L	CTSF
		ASPN	LTF	ELMOD1	DPYD
		ADH1C	KRT19	FABP4	TNNI1
		ECI1	OAT	PAPLN	UBL7

TNXB	CRP	Patr-A	CRYM
SERPINA3-7	CLU	FADD	MFAP2
PLVAP	TNXB	FAH	PSAT1
SERPINB2	VWF	IGLV3-1	DTYMK
BLVRA	ADAMTSL5	PCP4L1	GTPBP8
GPX4	MSRB2	PGPEP1	SNX24
AS3MT	GCHFR	PKIA	SNX24
ACSL3	Igkc	PKIA	JCHAIN
HEBP1	SELENOF	PKIA	UBL5
ADI1	SURF1	PKIA	SRGAP2
ITIH4	NAAA	GSTT1	PDSS2
PECR	--	SERPINA3-6	CHCHD5
APOH	PRKAG3	CFP	RNF181
LDHD	ECM1	IGHA1	CYP2F3
TXNRD1	SELENOM	METAP1D	Rbbp9
KLHL31	MCEE	LBP	HLA-A
GPX3	A1m	ATP23	LGALS3
PTGR1	IGLV2-14	STK39	IGLV2-14
PTER			
