# Supplementary material for: LncRNA and Protein Expression Profiles Reveal Heart Adaptation to High-Altitude Hypoxia in Tibetan Sheep
Source: Int J Mol Sci. 2023 Dec 27;25(1):385. doi: 10.3390/ijms25010385 (PMC10779337; doi:10.3390/ijms25010385)
Supplement: Supplementary file 1 [file ijms-25-00385-s001.zip › Table S3.pdf]

Table S3 Differentiation in lncRNA targets genes and DEPs involved with altitude hypoxia adaptation

Description	Pathway		
Metabolism of cofactors and vitamins	Retinol metabolism(ko00830)	DE lncRNA target genes	UGT2B31; UGT2C1; CYP4A6; RDH16; HSD17B6; BCO1; UGT1A3; **ADH1C**; ADH6; CYP2B4; CYP2B11; CYP2C18; CYP3A28; CYP3A24; CYP1A2; CYP1A1
		DEPs	ALDH1A1; **ADH1C**; AOX1
Cancer: overview	Chemical carcinogenesis - DNA adducts(ko05204)	DE lncRNA target genes	UGT2B31; UGT2C1; UGT1A3; CYP2E1; CYP2A13; SULT2A1; **ADH1C**; CBR1; ADH6; CYP2B4; CYP2B11; CYP2C18; GSTA1; CYP3A28; CYP3A24; CYP1A2; CYP1A1
		DEPs	GSTM5; **ADH1C**; HSD11B1; GSTT1
Lipid metabolism	Steroid hormone biosynthesis (ko00140)	DE lncRNA target genes	CYP2D14; UGT2B31; UGT2C1; HSD17B6; AKR1D1; HSD17B12-B; UGT1A3; **AKR1C1**; **PGFS**; CYP2E1; CYP2B4; CYP2B11; CYP3A28; CYP3A24; CYP1A2; CYP1A1
		DEPs	**AKR1C1**; **PGFS**; HSD11B1
Endocrine system	PPAR signaling pathway(ko03320)	DE lncRNA target genes	**APOA2**; FABP1; HMGCS2; CYP4A6; SLC27A2; PCK1; **FABP4**; CYP8B1; APOC3
		DEPs	**APOA2**; CPT1A; **FABP4**; ACSL3; FABP7; EHHADH; APOC3; APOA1; ACAA1; ACOX3
Xenobiotics biodegradation and metabolism	Metabolism of xenobiotics by cytochrome P450(ko00980)	DE lncRNA target genes	UGT2B31; UGT2C1; UGT1A3; CYP2E1; CYP2A13; SULT2A1; **ADH1C**; CBR1; ADH6; GSTA1; CYP1A2; CYP1A1
		DEPs	GSTM5; **ADH1C**; HSD11B1; GSTT1; CYP2F3
Digestive system	Cholesterol metabolism(ko04979)	DE lncRNA target genes	**APOA2**; ABCB11; APOC2; **APOH**; APOB; APOA4; **APOC3**
		DEPs	**APOA2**; TSPO; **APOH**; APOE; **APOC3**; APOA1; NPC1
Digestive system	Fat digestion and absorption(ko04975)	DE lncRNA target genes	FABP1; MTTP; APOB; **APOA4**
		DEPs	**APOA4**; APOA1
Biological processes	inflammatory response (GO:0006954)	DE lncRNA target genes	**APOA2**; KNG1; AHSG; **A1M**; C3; C5; C9; **CRP**; **LBP**; HP; KLKB1; TF; C8A; AOC3; AOC3; F2; VTN; **IDO1**; C3
		DEPs	**CRP**; **A1M**; **APOA2**; **LBP**; TFRC; THBS1; C4BPA; ORM1; ITIH4; CFP; HLA-DRB1; EPHX2; VNN1; FN1; GPX1; **IDO1**
Biological processes	Protein activation cascade (GO:0072376)	DE lncRNA target genes	KNG1; **A1M**; C3; C5; C9**; APOH**; **CRP**; MASP2; FGG; FGB; KLKB1; C8A; F2; **IGLV1-40**; C3
		DEPs	IGKC; **A1M**; **APOH**; VWF; CFD; F9; C4BPA; **CRP**; CLU; CFP; IGLV2-11; IGLV3-25; IGLV3-19; C1QC; **IGLV1-40**; IGLV2-14; IGLV3-1
Biological processes	Response to lipid (GO:0033993)	DE lncRNA target genes	**APOA2**; SLCO1B3; HMGCS2; CYP4A6; GC; FHL2; **FBP1**; **ALDOB**; **KRT19**; **AKR1C1**; **PGFS**; **CRP**; **LBP**; PAH; HP; PCK1; HMBS; SULT2A1; **ADH1C**; CBR1; TAT; ADH6; ARG1; **FABP4**; FOS; CDO1; HAVCR1; CYP3A24; CYP3A24; CCL3; ABCC2; CYP1A2; CYP1A1; TTR; GBP6

		DEPs	**KRT19**; TSPO; **APOA2**; PDXK; **ALDOB**; NASP; **FBP1**; **CRP**; GPX4; MGP; **LBP**; ACAT2; PRKCG; CST3; **ADH1C**; SPARC; GBP3; CPT1A; **PGFS**; TNC; **AKR1C1**; **FABP4**; CA2; PYCARD; THBS1; GSTM5; ORM1; S100B; CAT; PNMT; MAOB; HSPB6; CDK6; HSD11B1; GPX1; STAT1; FN1
Biological processes	Humoral immune response (GO:0006959)	DE lncRNA target genes	**A1M**; C3; C5; C9; **CRP**; MASP2; FGB; ZP2; C8A; **IGLV1-40**; TUBA8
		DEPs	IGKC; **A1M**; IGHA1; HLA-DRB1; CFD; IGLV3-19; C4BPA; **CRP**; CLU; CFP; IGLV3-1; IGLV3-25; **IGLV1-40**; C1qc; BOLA-DQB; IGLV2-14; IGLV2-11
Biological processes	Response to oxygen-containing compound (GO:1901700)	DE lncRNA target genes	**APOA2**; AHSG; FABP1; HMGCS2; **FBP1**; **ALDOB**; ACTC1; **AKR1C1**; **PGFS**; CDH1; **CRP**; HNF4A; **LBP**; NPPA; HP; PCK1; SULT2A1; **ADH1C**; ADH6; ARG1; **FABP4**; FOS; TF; CDO1; CCL3; GCGR; GBP6
		DEPs	**CRP**; TSPO; **APOA2**; **LBP**; **ALDOB**; PRKCG; **FBP1**; **ADH1C**; CA3; GBP3; CPT1A; HBB; TNC; CST3; **FABP4**; FBLN5; HSPB6; PYCARD; ACAT2; THBS1; **AKR1C1**; ORM1; SPARC; IGLV2-14; IGLV2-11; **PGFS**; ACTG2; PNMT; MAOB; HLA-DRA; STAT1; FN1; ACTA1; CAT; HBA1; GPX1; MYH11; APOM; HSD11B1
Biological processes	Immune effector process (GO:0002252)	DE lncRNA target genes	**APOA2**; **A1M**; C3; MX2; PGLYRP2; C5; C9; **IFI16**; **CRP**; **LBP**; **HPX**; MASP2; TUBB4A; C8A; CCL3; **CFHR2**; HLA-DMB; **IGLV1-40**; C3
		DEPs	IGKC; **A1M**; **APOA2**; **LBP**; LGALS1; MX1; C4BPA; **CRP**; SAMHD1; GBP3; FADD; **HPX**; **IFI16**; PGLYRP1; CLU; S100B; HLA-DRB1; CFD; RFTN1; C1QC; CD74; IGHA1; PYCARD; IGLV3-25; STAT1; MPO; LGALS3; CFP; **CFHR2**; BOLA-DQB; IGLV2-14; IGLV2-11; **IGLV1-40**; IGLV3-1; IGLV3-19
Biological processes	Regulation of cell proliferation (GO:0042127)	DE lncRNA target genes	KNG1; FABP1; **AKR1C1**; **PGFS**; CPB2; CDH1; **APOH**; NAP1L1; FAP; LRG1; HPN; ARG1; **FABP4**; **RGN**; TF; **ICA**; CCL3; F2; KLB; **IDO1**; CXCR3; HLA-DMB; **ABCC4**
		DEPs	**FABP4**; TSPO; OGN; PRKCG; SPTA1; RIDA; CST3; GPX1; CLU; **PGFS**; TNC; ECM1; TFF2; **AKR1C1**; CD74; **RGN**; THBS1; **APOH**; FADD; COL18A1; LGALS3; PRKRA; CAMK2A; **ABCC4**; HLA-DRB1; CD109; SPARC; **IDO1**; LAMB1; S100B; SPARCL1; CASK; PYCARD; CDK6; IGHA1; ADK; **ICA**; LTF; STAT1
Biological processes	Reactive oxygen species metabolic process (GO:0072593)	DE lncRNA target genes	**CRP**; HP; **RGN**; CYP1A2; CYP1A1
		DEPs	**CRP**; FBLN5; HBB; CAT; MAOB; GPX1; HBA1; GCHFR; GPX4; **RGN**; MPO; TXNRD1; GPX3
