# Supplementary material for: LncRNA and Protein Expression Profiles Reveal Heart Adaptation to High-Altitude Hypoxia in Tibetan Sheep
Source: Int J Mol Sci. 2023 Dec 27;25(1):385. doi: 10.3390/ijms25010385 (PMC10779337; doi:10.3390/ijms25010385)
Supplement: Supplementary file 1 [file ijms-25-00385-s001.zip › Table S4.pdf]

Table S4 Differential expression LncRNAs in profile 5 and profile 7 of trend clustering

Pattern	DE LncRNA			
Profile 3	ENSOART00020002796	ENSOART00020016070	MSTRG.11195.1	MSTRG.16347.1
	ENSOART00020003573	ENSOART00020016122	MSTRG.11772.1	MSTRG.16626.2
	ENSOART00020003962	ENSOART00020024749	MSTRG.1193.1	MSTRG.16647.1
	ENSOART00020004609	ENSOART00020027412	MSTRG.12014.1	MSTRG.16732.1
	ENSOART00020005091	ENSOART00020029824	MSTRG.12379.1	MSTRG.17129.1
	ENSOART00020006729	ENSOART00020031167	MSTRG.12489.1	MSTRG.1877.1
	ENSOART00020007265	ENSOART00020036492	MSTRG.1341.1	MSTRG.2891.2
	ENSOART00020008220	ENSOART00020040698	MSTRG.14115.1	MSTRG.4037.2
	ENSOART00020008223	MSTRG.10026.1	MSTRG.14115.2	MSTRG.7424.1
	ENSOART00020008273	MSTRG.1100.10	MSTRG.15339.1	MSTRG.983.1
	ENSOART00020013655	MSTRG.1100.8	MSTRG.16222.3	MSTRG.983.2
Profile 7	ENSOART00020002028	ENSOART00020029432	MSTRG.10631.1	MSTRG.4183.2
	ENSOART00020006963	ENSOART00020029670	MSTRG.11593.2	MSTRG.5153.2
	ENSOART00020010355	ENSOART00020030440	MSTRG.11593.3	MSTRG.5765.2
	ENSOART00020013887	ENSOART00020038924	MSTRG.13236.1	MSTRG.5938.1
	ENSOART00020013993	ENSOART00020040396	MSTRG.3416.1	MSTRG.7916.4
	ENSOART00020019309	MSTRG.10045.1	MSTRG.362.1	

analysis
