# Supplementary material for: LncRNA and Protein Expression Profiles Reveal Heart Adaptation to High-Altitude Hypoxia in Tibetan Sheep
Source: Int J Mol Sci. 2023 Dec 27;25(1):385. doi: 10.3390/ijms25010385 (PMC10779337; doi:10.3390/ijms25010385)
Supplement: Supplementary file 1 [file ijms-25-00385-s001.zip › Table S5.pdf]

Table S5 Differential expression Proteins in profile 5 and profile 7 of trend clustering analysis

Pattern	DEPs			
Profile 3	NDUFAF7	TNXB	MAP1LC3A	EBP
	UBL5	CTSF	MFAP2	GPX1
	S100A1	Ntn4	ADH1C	SERPINB2
	ASPN	THBS1	Srm	Rbbp9
	PSAT1	CTSD	AOX1	OAT
	PTP4A1	TPP1	GPX4	FAH
	HEBP1	FUOM	TMEM109	SMG1
	RECK	CLU	FAM53C	ITIH4
	PGPEP1	ELMOD1	LGALS3	KLHL31
	DPY19L1	SERPINA3-7	ACSM5	PECR
	TNC	LBP	ADAMTSL5	NPC1
	SELENOF	CYP2F3	ACOX3	AK4
	SDR39U1	PAPLN	CRYM	PPT1
	PRXL2B	PDSS2	ATP23	SAA4
	SELENOO	AOC1	SELENOM	ECI1
	TNXB	Patr-A	RBM3	
Profile 5	MYLK	LGALS1	SERPINA3-8	PACSIN1
	S100A4	MYL9	ITGA3	SMTN
	OGN	PGFS	EPHX2	LIPE
	ASS1	MSRA	MYOT	Aldh1l1
	EHHADH	CNN1	CAT	ACTA1
	FBP1	COL18A1	TKFC	MAOB
	ALDOB	DPT	TXNRD1	TACO1
	CSRP2	Gstm5	PDLIM7	HSD11B1
	ACTN1	KRT18	GPX3	CD109
	TAGLN	INPP1	PSPH	CPS1
	PRELP	KRT8	TEX2	FLNA
	NAAA	MFGE8	PDLIM3	Lmod1
	HBB	LUM	SOD3	CLYBL
	CP	ACTG2	VNN1	FKBP5
	PODN	CRP	VNN1	CSRP1
	KRT19	THA2	FBLIM1	MYH11
	Echdc3	LECT2	CKB	MUSTN1
	APOE	COL12A1	FABP4	BGN
	ACAT2	PTGIS	ORM1	ACAA1
	AKR1C1	SERPINA3-3	THEM4	ATP6V0C
	ANGPTL2	CES1	F9	SHMT1
	MGRN1	HPX	RGN	ANXA8
